# Post-rehabilitation self-management support on physical activity and nutrition, including mHealth, improves physical capacity, physical activity, and health related quality of life in people with Parkinson’s– results from a randomised controlled trial

**DOI:** 10.1186/s12966-026-01888-y

**Published:** 2026-02-17

**Authors:** Sigrid Ryeng Alnes, Ellisiv Laerum-Onsager, Asta Bye, Annette Vistven, Erika Franzén, Mette Holst, Therese Brovold

**Affiliations:** 1 Department of Rehabilitation Science and Health Technology, Faculty of Health Science, OsloMet - Oslo Metropolitan University, Oslo, Norway; 2 Department of Nursing, Lovisenberg Diaconal University College, Oslo, Norway; 3 Department of Haematology, Oslo University Hospital, Oslo, Norway; 4 Department of Nursing and Health Promotion, Faculty of Health Science, OsloMet - Oslo Metropolitan University, Oslo, Norway; 5 European Palliative Care Research Centre, Department of Oncology, Oslo University Hospital, Oslo, Norway; 6 Institute of Clinical Medicine, University of Oslo, Oslo, Norway; 7 Unicare Fram Rehabilitation centre, Rykkin, Norway; 8 Division of Physiotherapy, Department of Neurobiology, Care sciences and Society, Karolinska Institutet, Stockholm, Sweden; 9 Medical Unit Allied Health Professional, Theme Women’s Health and Allied Health Professionals, Karolinska University Hospital, Stockholm, Sweden; 10 Nutrition Research Unit, Center for Nutrition and Bowel Disease Intestinal Failure, Aalborg University Hospital, Aalborg, Denmark; 11 Department of Clinical Medicine, Aalborg university, Aalborg, Denmark

**Keywords:** Self-management support, MHealth, Parkinsons disease, Nutrition, Exercise, Activity tracker

## Abstract

**Background:**

Maintaining long-term engagement in physical activity (PA) and following nutrition recommendations after rehabilitation is challenging for people with Parkinson’s. Sustained behavioural change requires more than initial education; person-centred, self-management support may be key to maintain health-promoting routines. However, structured follow-up is often lacking. Digital health interventions, including mobile health (mHealth), offer scalable solutions to provide ongoing support beyond rehabilitation. This study aimed to examine the effect of an individualised, mHealth support programme targeting self-management of PA, and nutrition on physical capacity, nutritional status, HRQOL, Physical function and engagement in PA in people with Parkinson’s.

**Methods:**

A single-blind, two-arm randomised controlled trial evaluating the effects of a six-month individualised mHealth self-management support programme on physical capacity and related outcomes in people with Parkinson’s following inpatient interdisciplinary rehabilitation. Participants were randomly assigned (1:1) to an intervention group (mHealth); monthly remote consultations plus activity tracker, or a control group; usual care. The primary outcome was physical capacity (6MWT). Secondary outcomes were nutritional status (PG-SGA SF), Health Related Quality of Life (PDQ-39), physical function and engagement in PA (self-reported PA and activity tracker data). Linear mixed models for repeated measures were used to assess group differences over time.

**Results:**

A total of 100 participants (40% female, mean age 67,5 years) were randomised (50 per group). At six months we observed significant between-group differences on 6MWT in favour of the intervention group (mean: 33.1 m; 95% CI: 14.8 to 51.3; *p* < 0.001; effects size = 0.75). Significant between-group differences were also observed in PDQ-39 SI(mean: -6.1; 95% CI: -9.5 to -2.8; *p* < 0.001; effect size 0.93) and in physical activity frequency (*p* = 0.02; effect size = 0.51). Additionally, the mHealth group significantly increased their daily steps (*p* = 0.006) and weekly intensity minutes (*p* = 0.042). No significant differences were found for nutritional status, or physical function.

**Conclusions:**

The six-month mHealth self-management support programme improved physical capacity, Health Related Quality of Life, and physical activity in people with Parkinson’s post-rehabilitation. These findings highlight the potential of scalable, person-centred digital self-management support interventions to sustain health-promoting behaviours beyond clinical settings.

**Trial registration:**

The study was registered on ClinicalTrials under the code NCT04945876: https://clinicaltrials.gov/expert-search?term=NCT04945876. First registration March 1, 2021.

**Supplementary Information:**

The online version contains supplementary material available at 10.1186/s12966-026-01888-y.

## Background

 Parkinson’s affects over 10 million people worldwide and is rapidly increasing [1]. Within five years of disease onset, up to 50% experience motor- and non-motor symptoms [2] affecting daily functioning, nutritional status [3, 4], cognition [5], and health-related quality of life (HRQOL) [6]. Interdisciplinary rehabilitation combining physical activity, and nutritional interventions alongside pharmacological treatment are an essential strategy that improve both motor and non-motor outcomes in Parkinson’s [7–11].

Maintaining long-term participation in health-promoting behaviours, such as regular leisure-time physical activity(PA), including structured exercise [12], and following dietary recommendations, is essential for achieving rehabilitation goals in Parkinson’s [13]. However, sustaining these behaviours requires significant effort and is challenging over time, and factors such as low self-efficacy, poor outcome expectations, pain, and fear of falling often undermine continued engagement [14–17]. Understanding these challenges requires recognising the interplay between individual, physical, and environmental factors [15]. Consequently, improving engagement requires more than prescribing an intervention, it requires addressing these barriers and fostering sustained self-management. Self-management encompasses the daily tasks of living with a chronic condition and the knowledge, skills, and confidence needed to perform them effectively. It is an active process of managing one’s health, making informed decisions, and adapting to challenges to promote well-being and quality of life [18–22]. While self-management is typically given priority and focus during rehabilitation, many people with Parkinson’s struggle to maintain health-promoting behaviours once formal programmes end [23–25].

A personalised approach that integrates self-management support post-rehabilitation may facilitate sustained health-promoting behaviours [15, 26–30]. Self-management support is a person-centred, collaborative strategy that helps individuals build essential self-management skills tailored to their preferences, needs and capabilities [30–33]. Self-management support has been found to improve HRQOL, self-management behaviour and overall functioning across patient groups [30], and is strongly emphasised by people with Parkinson’s and their caregivers [34–36]. However, access to ongoing self-management support is often limited due to geographical disparities and resource limitations, resulting in inconsistent care availability [34, 35].

Digital health solutions, such as mobile health (mHealth), offer a scalable, and accessible approach for self-management support [37–41]. mHealth involves smartphones and wearable devices, which can increase motivation [42], provide real-time activity feedback [43], and promote sustained engagement to PA [44, 45]. While these benefits are promising, most research has focused on mHealth solutions targeting single components, typically PA. Effects of more comprehensive mHealth support programmes on nutrition, physical capacity, HRQOL, and self-management remain unclear [30]. To address this, we developed an mHealth programme providing monthly individualised support via video or phone, focusing on PA, nutrition and related issues, alongside the use of an activity tracker. To our knowledge, no study has examined the effect of such a programme on PA, nutritional status, and HRQOL in people with Parkinson’s after in-patient rehabilitation.

The intervention is grounded in Lorig and Holman’s [19] self-management framework, which identifies core self-management skills as problem-solving, decision making, resource utilisation, partnership with health-care providers, action planning and self-monitoring. Furthermore, the intervention integrates self-management support elements from Dineen-Griffin et al. [30], including tailored patient-provider consultations, feedback, personalised action plans, symptom monitoring, and stress-management.

This study aimed to examine the effect of an individualised, mHealth support programme targeting self-management of PA, and nutrition, on physical capacity in people with Parkinson’s after interdisciplinary rehabilitation. Secondary aims included effects on nutritional status, HRQOL, Physical function and engagement in PA.

We hypothesised that participants receiving the intervention would show significant improvements in PA engagement, nutritional status, physical capacity, physical function, and HRQOL at 3 and 6 months compared to controls.

## Methods

### Design

A single-blinded, two-group, pragmatic randomised controlled trial [46].

### Setting and participants

All participants were recruited during the final week of a 4–5-week elective, interdisciplinary inpatient rehabilitation programme at a rehabilitation centre in Norway. The centre offers specialist Parkinson’s rehabilitation and operates under a contract with the national health services. Referrals are made by general practitioners or neurologists, with placements allocated by municipalities. The programme admits individuals at various disease stages, provided they are motivated and have sufficient physical and cognitive capacity to actively participate in and benefit from the rehabilitation. Although most are inpatients, a small number attend as outpatients.

The rehabilitation programme was based on current clinical guidelines on the treatment of Parkinson’s, such as the European guidelines covering physiotherapy and nutrition, among other areas [47]. It focused on enhancing self-management through a range of activities, including education on topics such as PA, exercise and nutrition, and opportunities to try out various exercise modalities [26, 48]. As part of the rehabilitation programme, participants were offered an individually tailored home-based exercise plan and were encouraged to review the digital resource ‘Eating smart online’(Matvett på nett), an e-learning course on nutrition for people with Parkinson’s [49]. Further details about the rehabilitation programme are provided in the published protocol and in Appendix 1 [46].

Eligible individuals were recruited by a research assistant (PT, AV) during the final week of their stay at the rehabilitation centre and all participants gave informed consent before participation in the study. The intervention group (mHealth group) were offered a six-month individually tailored mHealth support programme after discharge (described below). This intervention was delivered remotely to participants in their own homes. The control group received usual care (described below) except for re-testing.

The inclusion criteria: > 40 years, idiopathic Parkinson’s, a Hoehn & Yahr (H&Y) stage of 1–3, living at home within a maximum travel time of 2.5 h from the rehabilitation centre, where follow-up testing were done, and owning a smartphone.

Exclusion criteria: Assessed by a doctor as not able to tolerate exercise, dementia diagnosis or severe dysphagia, people receiving enteral or parenteral nutrition. Furthermore, to enhance the efficacy of intervention in comparable groups requiring intervention, those who exercised regularly more than twice a week before the rehabilitation stay were also excluded.

### Randomisation, assessment, and blinding

Participants were allocated to either the intervention group (mHealth group)(*n* = 50) or the control group (usual-care group)(*n* = 50) at a 1:1 ratio using a computer-generated, permuted block randomisation scheme after informed consent and baseline assessment. All assessments (at inclusion, after three and six months) were conducted at the rehabilitation centre by a trained assessor blinded for group allocation. Baseline testing was conducted at the final day of the rehabilitation stay.

Intervention.

### The mHealth group

The mHealth intervention was designed to support people with Parkinson’s in maintaining engagement in PA, physical function, nutritional status, and HRQOL after rehabilitation. It is described according to the TiDier checklist (Appendix 2). See Table 1 and the published protocol [46] for more details on the intervention description.

**Table 1:** Description of the mHealth intervention

Intervention component	Description	Details and examples
Initial consultation	A face-to-face consultation with the follow-up physical therapist (PT) at the rehabilitation centre prior to discharge to introduce the intervention, establish a partnership with the PT, and set up the activity tracker.	Participants received information about the intervention and had the opportunity to ask questions. The PT demonstrating the use of and how to interpret pulse data and intensity minutes, log and review activity record on the Garmin Vivosmart 4 activity tracker. Scheduling the first remote follow-up by video/phone.
First remote follow-up session	A video or phone session (up to 60 min) within two weeks after discharge. The goal was to build partnership with the follow-up PT, discuss goals and actions plans, and provide support.	Topics: Repetition of the information on the intervention and activity tracker. Conversations about participants experiences, feelings, and motivation for leisure-time physical activity (PA), nutrition, medication, sleep, life situation, and other relevant topics. Short and long-term goals and action planning were discussed regarding PA, exercise and nutrition(if relevant). The participants and the PT discussed types of physical activities and potential barriers and solutions for following up on action plans.
Subsequent monthly remote follow-up sessions	Five to six subsequent remote sessions (up to 60 min) were scheduled to provide structured, person-centred self-management support. Each new session was scheduled at the end of the previous one.	Subsequent sessions were guided by participants’ priorities and followed up on relevant themes from the last session. The PT and participant evaluated progress, goals and plans. What had worked well? What had been challenging, and why? Goals and plans were adjusted accordingly to overcome relevant barriers. Examples of goals: - Being able to walk a specific trail by summer - Completing a hill walk without stopping - Playing all holes on a golf course - Meeting weekly intensity-minute target - Exercising three times a week Examples of action plan adjustments - A participant struggles to reach targeted heart rate on walks -> PT recommends adding hills or stairs to the walk - A participant finds their gym program boring -> PT helps explore alternative exercises or activities - Poor sleep affects exercise ability -> PT reviews sleep routines and suggest strategies to improve sleep - A participant is having trouble finding time for PA - PT helps review their weekly schedule to identify opportunities to prioritise and make time for their desired PA. - A participant struggles with energy management. The PT helps them discuss and develop a conscious strategy for prioritising weekly tasks. - A participant experiences frequent nighttime urination (no medical cause indicated) -> PT recommend shifting fluid intake to the morning and afternoon Nutrition was discussed in every session, including appetite and weight changes. The PT identified any changes in need of follow-up, suggested practical dietary adjustments, and referred to the GP if medication review or further assessment was required.
Activity Tracker	Participants were provided with a wrist-worn activity tracker to support self-monitoring and self-regulation of daily PA.	Participants were encouraged to actively monitor daily steps and activity levels. Tracker data such as step count, weekly intensity-minute goals, and pulse targets were reviewed during consultations to reflect on progress
Text-message(SMS)-support	Participants could contact the PT via SMS with any questions between sessions.	The PT responded by SMS, a brief phone call, or during the next scheduled session, depending on the nature of the question. Examples include newly emerged symptoms, pain or issues with the activity tracker.
Nutritional follow-up	Participants in nutritional-risk received individualised guidance from a nutrition specialist.	Guidance included personalised recommendations, such as increasing energy intake if necessary, adjusting protein distribution in relation to levodopa use, and increasing the intake of dietary fibre. The PT was present during the consultation and later reinforced and followed up on these recommendations.
Documentation & continuity	All consultations were recorded in a secure system to ensure continuity of care throughout the six-month intervention.	The PT referred to notes from previous sessions to follow-up on prior decisions and activities, for example: “ last time, you decided to focus on x, or you were to try out y. How did that go?”.

The intervention started upon discharge from the rehabilitation centre and lasted for six months. It included monthly remote consultations (video or phone, up to 60 min) with a follow-up physical therapist (PT, SRA) and the use of a Garmin Vivosmart 4 activity tracker. Before discharge, participants had a face-to-face session with the PT at the rehabilitation centre reviewing programme information and learn to use the activity tracker for self-monitoring and motivation.

Post rehabilitation, participants engaged in six to seven monthly remote consultations. The first session (within two weeks) focused on reviewing PA and nutritional habits, co-creating goals, and revising individualised action plans. Action plans primarily targeted PA but could also include targets such as sleep routines, hydration, and symptom management (e.g. managing freezing). Subsequent sessions were participant led, reflecting on progress, barriers for following the action plans, and develop strategies to overcome them. The PT referred participants to other healthcare personnel when appropriate.

Nutritional status was addressed in every session, including appetite, weight loss, and nutritional impact symptoms. Those identified as at-risk for malnutrition received digital individualised nutritional guidance from a specialist. These sessions were tailored using baseline assessment, three-day dietary records, and participants` self-reported nutritional concerns.

Guidance from the PT was based on current clinical guidelines covering relevant areas such as PA, nutrition, sleep, and symptom management. The intervention employed a person-centred and community-oriented approach to integrate rehabilitation habits into daily routines and connect participants with community resources (e.g. local gyms, group classes or ParkinsonNet physiotherapists) [50].

Sessions emphasised self-management skills providing individualised feedback to build confidence through short-term goals and new routines. The activity tracker facilitated self-monitoring of PA, with personal motivators (e.g. preferred exercise types, social support and gamification elements) incorporated when relevant (see Appendix 3 for framework alignment).

Between sessions participants could contact the PT for questions via mobile text message (SMS). Each consultation was documented in logbooks to inform subsequent follow-ups and ensure continuity.

### Usual-care group

Before discharge, participants got a face-to-face conversation at the rehabilitation centre with the follow-up PT, during which they received information on re-testing and were encouraged to follow the recommendations on PA, nutrition and other relevant areas from the rehabilitation centre and review the digital resource ‘Eating smart online’. In line with usual care, participants received no further instructions after returning home except for re-testing.

### Education of intervention deliverers

A PT, with an MSc in physical therapy and experience in counselling and Parkinson’s, delivered all follow-up sessions using a semi-structured manual developed by the research team (see published protocol [46]). Following Dineen-Griffin et al.’s [30] recommended components for self-management support, the PT applied core communication skills such as active listening, encouragement, empathy, and clear guidance to ensure supportive consultations. Participants identified as being at nutritional risk received individualised follow-up from a registered nurse(RN, ELO) with a PhD in nutrition or a nutritionist (AB).

### Outcomes and assessments at baseline and 3- and 6 months follow-up

During baseline assessment, data on participants’ health and demographic characteristics were collected, including age, gender, living arrangements, education level, work status, previous falls, use of home care services, PA levels, prior PT follow-up, medication list, comorbidities, and time since Parkinson’s diagnosis.

Baseline measures also included Cognitive function (Montreal Cognitive assessment, MoCA [51], and all tests on physical capacity, physical function, nutritional status, body composition, and HRQOL.

At 3 months, participants were reassessed for physical capacity, physical function, nutritional status, body composition, and HRQOL.

At 6 months, previous falls, PA levels, medication changes, work status, use of home-care services, and PT follow-up were recorded. Participants also repeated measures on physical capacity, physical function, nutritional status, body composition, and HRQOL. Additionally, they rated the usefulness of the digital follow-up and activity tracker on a 1 (not useful) to 5 (very useful) scale.

All assessments were conducted at same time of day to control for medication effects. Participants completed all questionnaires independently, with the assessor was available for questions.

### Physical capacity, function and activity

The primary outcome was physical capacity measured with the 6-minute walk test (6MWT) which is recommended for assessing physical capacity in people with Parkinson’s [47, 52]. The test has good clinical utility, responsiveness, and validity for the Parkinson’s population [52] and measures the distance (meters) a person can walk within six minutes. Although a PD specific MCID is not established, a systematic review suggests that a change of 14.0 to 30.5 m can be considered clinically significant in adults with various pathologies [53].

Balance was measured by the Mini Balance Evaluation Systems Test (Mini BESTest) comprising 14 items with a total score between 0 and 28, a low score indicates reduced balance [54]. The Five Times Sit to Stand test (FTSST) was used to measure functional mobility. During the test, the participant is asked to, without the use of their arms, stand up and sit down five times as quickly as possible [55].

Leisure-time PA, including exercise was self-reported at baseline and at six months. The question was ‘How often do you exercise? (by exercise we mean activities such as taking a walk, cross-country skiing, cycling, swimming or other types of exercise or sports activities.), with response categories (scores in parentheses): ‘Never’ (0.0), ‘Less than once a week’ (0.5),‘Once a week’ (1), ‘2–3 times a week’ (2.5) and ‘Nearly every day’ (5).The validated question was derived from a longer questionnaire used in the HUNT study in Norway [56]. Other components of the questionnaire, such as intensity and duration, were not included in this study.

For the mHealth group, data from the activity tracker were gathered on daily steps, hearth rate (HR) and weekly intensity minutes. Intensity minutes were logged as either moderate (70–80% of maximum HR) or vigorous (80–100% of maximum HR). Maximum HR was automatically calculated by the activity tracker based on the formula 220 minus age [57]. The Garmin Vivosmart 4 activity tracker is a wrist-born activity tracker that is found to be reliable for step-counting in people with Parkinson’s [43, 58].

### Nutritional status

Overall nutritional status was assessed with the Patient-Generated Subjective Global Assessment Short form (PG-SGA SF) [59]. The questionnaire tracked weight, food intake, symptoms affecting intake, and physical function to evaluate nutritional status. Scores were calculated per the PG-SGA SF protocol: 0–1 indicate well-nourished, 2–3 required education, 4–8 warranted dietitian intervention, and ≥ 9 indicated critical symptom management. Participants scoring ≥ 4 were, according to the PG-SGA SF protocol, considered as in need of intervention and received targeted follow-up [59]. The TANITA BC-418 scale was used to measure weight [60, 61]. Body mass index (BMI) was calculated using measured weight and self-reported height and classified per WHO guidelines [62].

### HRQOL

HRQOL was assessed using the Parkinson’s Disease Questionnaire (PDQ39) [63], which comprises 39 items across eight dimensions: mobility, daily activity, emotional well-being, stigma, social support, cognitions, communications and bodily discomfort [64]. Responses are on a 5-point scale (0 = never to 4 = always), with scores calculated for each and summarised in the PDQ-39 Summary Index (PDQ-39 SI). Scores range from 0 to 100, where lower scores indicate better HRQOL [64]. The MCID for PDQ39 SI is considered to be -4.72 and + 4.22 [65].

### Adverse events

Participants in both groups were instructed to report any adverse events such as falls, pain, or injuries related to leisure-time activities at the follow-up testing sessions. Participants in the mHealth group also reported events during their monthly consultation.

### Sample size

Sample size was calculated from the primary outcome, 6MWT, assuming a between-group minimal detectable change (MDC) of mean (SD) 50 (80) meters. As no PD-specific between-group MDC is established, this conservative estimate was derived from previous studies among older adults with chronic conditions, including neurological disorders [66]. With a power of 80% and significance level of 0.05, the sample size was calculated to be sufficient with 41 participants in each group, 82 in total. We included 100 participants in total, to allow for possible loss to follow-up of approximately 20%.

### Statistical analyses

Statistical analyses were conducted using the IBM SPSS Statistics (v 29). A p-value of > 0.05 was considered statistically significant. Analysis was undertaken according to the intention to treat (ITT) principle. Continuous variables are described as means and standard deviations (SD) if normally distributed and median and 1st quartile and 3rd quartile (Q1,Q3) if skewed. Categorical variables are presented as proportions and percentages. The primary analysis for the between-group difference in 6MWT at 6 months was performed using a linear mixed model for repeated measures with a subject specific intercept, adjusted for baseline values. The type of treatment (intervention or control), baseline values, time and group were modelled as fixed effects. Results from this model are reported as the adjusted mean difference with its 95%CI and *p*-value. Weekly PA was derived by summarising participants frequency score [67]. Between-group differences in changes in PA frequency at 6 months were calculated using independent t-test. Within-group changes in daily steps and intensity minutes were calculated using the Wilcoxon’s signed rank test. Effect size(Cohen’s d) was calculated and interpreted according to the guidelines proposed by Cohen [68]; 0.2 = small effect, 0.5 = medium effect and 0.8 = large effect.

## Results

### Flow of participants

**Fig. 1:**
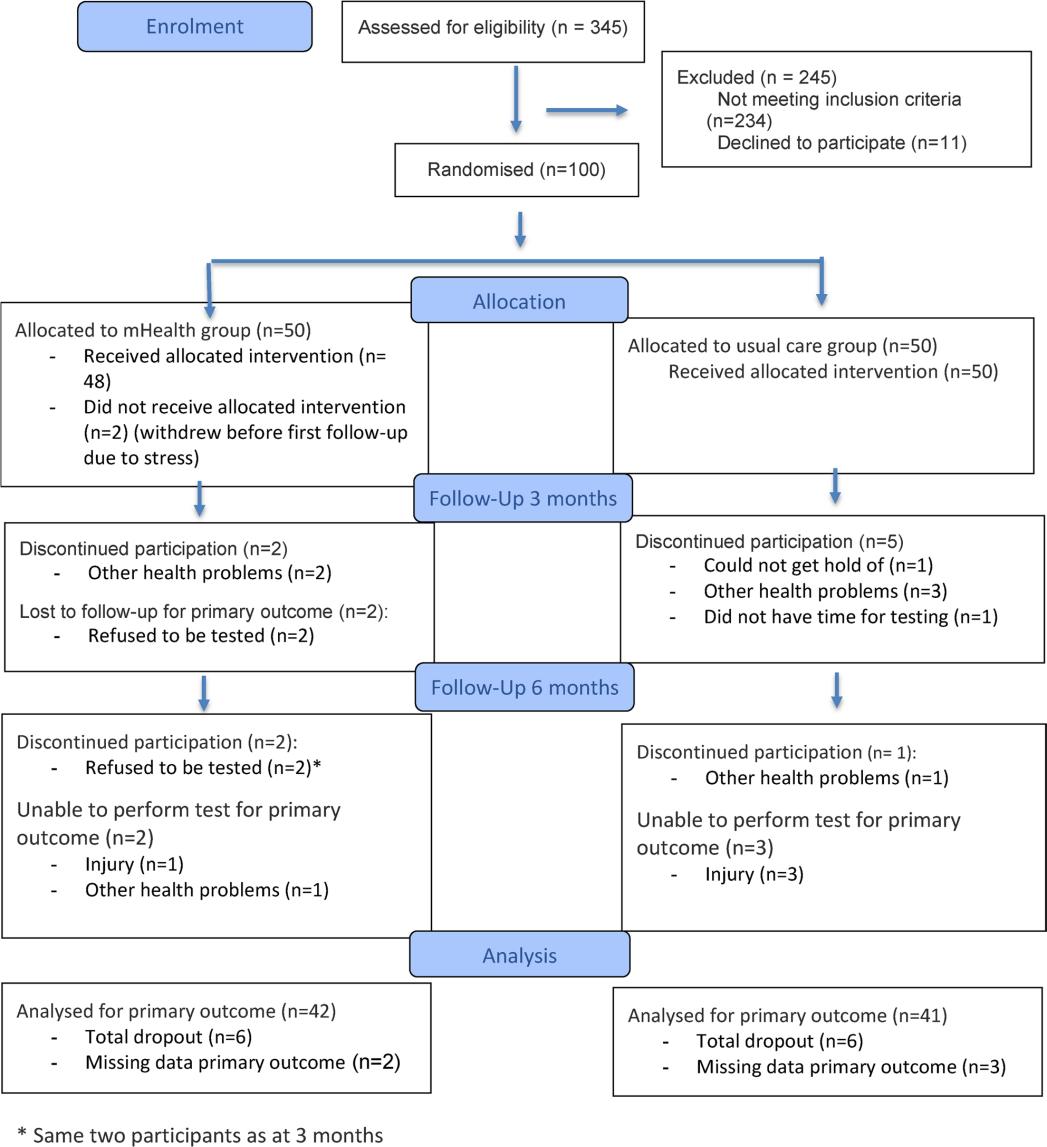
The Consort flow diagram [69]

Between April 2021 and January 2023, 345 patients were screened for eligibility. The main reason for exclusion was H&Y stage > 3 and living to far from the rehabilitation centre. A total of 111 patients were deemed eligible, of whom 11 declined participation. The remaining 100 consenting participants were randomised to either the mHealth group (*n* = 50) or the usual-care group (*n* = 50) (see Fig. 1). We found no statistically significant differences in age, gender, or disease severity between individuals who declined participation and those included in the study. Follow up testing was completed in June 2023.

We had a drop-out rate of 12% (*n* = 12) at six-months, equally distributed between groups. Noo statistically significant differences in baseline characteristics (such as age, gender and H&Y status) or outcome measures were observed between completers and dropouts. In addition, five participants were unable to conduct all physical function tests at follow up (Fig. 1). No adverse events related to the interventions were registered.

### Participants characteristics

Baseline characteristics of the total sample, and for the mHealth group and control group separately, are presented in Table 2. The mean (SD) age of participants was 67.5 (9.5) years, 60% were men and 92% had higher education. Seventy-six participants had H&Y stage between 2 and 3, and years since diagnosis was median: 4 (Q1,Q3: 1,7). MoCA score was median: 28 (Q1,Q3: 26,29). PG-SGA SF scores were median: 1 (Q1,Q3: 0,2), indicating a good nutritional status.

**Table 2:** Baseline characteristics of participants

Characteristic	Total (*N* = 100)	follow-up group (*n* = 50)	Usual care group *(n* = 50)
Age in years, mean (SD)	67.5 (9.3)	68.8 (9.6)	66.2 (9.0)
Female, *n* (%)	40 (40)	18 (36)	22 (44)
Bodyweight kg, mean (SD)	78.2 (16.7)	79.5 (17.7)	76.9 (15.8)
Body mass index, median (25,75 quartiles)	24.2 (22.1, 27.9)	24.5 (22.4, 28.3)	23.7 (22.1, 27.7)
Living alone, *n* (%)	27 (27)	15 (30)	12 (24)
Hoehn & Yahr stage^a^, *n* (%)	92 (92)	47 (94)	45 (90)
No other primary diagnoses, *n* (%)	27 (27)	29 (58)	34 (68)
Higher education^b^, *n* (%)			
stage 1–1.5	24 (24)	13 (26)	11 (22)
stage 2–3	76 (76)	37 (74)	39 (78)
years since diagnose, median (25,75 quartiles)	4 (0–18)	4 (0–18)	4.5 (0–18)
Cognition (MOCA), n= 91^c^, *n* (%)	28 (27,29)	28 (26,29)	29 (28,29)
occupational status, *n* (%)			
Currently working	14 (14)	3 (6)	11 (22)
Sick leave/disabled	23 (23)	11 (22)	12 (24)
Retired	63 (63)	36 (72)	27 (54)
Fallen last three months, *n* (%)	24 (24)	12 (24)	12 (24)
Medication, *n* (%)			
Only levodopa	33 (33)	21 (42)	14 (28)
Combination	62 (62)	28 (56)	32 (64)
Seeing a PT regularely, *n* (%)	49 (49)	23 (46)	26 (52)

MOCA: Montreal Cognitive Assessment. PT: Physiotherapist

^a^ Hoehn & Yahr: scale from 1–5, 1 is less affected (4–5 excluded from study)

^b^ College or university

^c^ missing data

### mHealth group

Forty-four participants (88%) completed all six, monthly support sessions and 41 deemed the follow-up as useful or very useful. 54% of participants used video-calling whilst 46% preferred to talk on the phone. The conversations lasted for 50 min on average, with longer sessions at the start of the intervention and shorter towards the end.

All conversations included discussions on motivation and PA, and participants reported activities since their last session, evaluated progress, explored motivational barriers and necessary adjustments. Nutritional discussions were tailored to individual needs. Common themes included: challenges maintaining exercise intensity; sleep disturbances and their impact on activity; Parkinson’s -related symptoms (pain, constipation, incontinence) limiting activity and participation; meal planning around medication; and managing daily energy balance between PA, daily tasks, and social commitments. Participants directed conversations toward their priority concerns.

Eleven participants (23%) needed follow-up from the nutritional specialist. Five of them were allocated based on their PG-SGA SF score at baseline, whilst the last six were referred to the RN by the PT, due to change in situation or symptoms that emerged in conversations. All participants in need of nutritional follow-up performed a 3-days dietary registration and received up to two hours of individualised nutritional advice from the nutrition specialist.

Thirty-six participants (72%) used the activity tracker for median 23 weeks (intervention duration: 26 weeks) and 64% evaluated the tracker as useful or very useful. Reasons for not using the activity tracker were undue stress, lack of ability (e.g. vision, tremor, or cognitive issues), or technical knowhow, reported during consultations. The decision not to use the activity tracker was made collaboratively by the participant and PT.

### Change after 6 months

The results from the linear mixed model analyses are presented in Table 3. At 6-month follow-up, the mHealth group showed a statistically significant improvement in the primary outcome 6MWT, compared to the control group. The between-group difference at 6-months, adjusted for baseline values, was mean: 33.1 m; 95%CI: 14.8 to 51.3; *p* < 0.001; effect size(Cohen’s d) = 0.75 indicating a clinically meaningful change. This between-group difference reflects a significant group x time interaction, where the mHealth group showed improvement overtime, whereas the control group demonstrated a significant decline.

**Table 3:** Effect of the Intervention

Variable	mHealth group (n = 50)			Usual care group (n =50)			Between group differences at 6 months		
	Baseline (n = 50) median (Q_1_, Q_3_)	3 month change Mean (95% CI) ^[n]^*	6 month change Mean (95% CI) ^[n]^*	Baseline (n = 50) median (Q_1_, Q_3_)	3 month change Mean (95% CI) ^[n]^*	6 month change Mean (95% CI) ^[n]^*	mean (95%CI)	*p*-value	effect size
Physical capacity (6MWT) (m)	549 (458, 617)	6.8 (-3.6 to 17.2)^44^	16.0 (2.8 to 29.3)^42^	570 (493, 638)	-7.6 (-21.7 to 6.5)^45^	-17.5 (-32.3 to -2.8)^41^	33.1 (14.8 to 51.3)	<0.001	0.75
MiniBESTest	26 (24, 27)	-0.03 (-0.7 to 0.6)^44^	-0.29 (-1.1 to 0.6)^43^	26 (24, 28)	-0.3 (-8.7 to 0.3)^44^	-0.6 (-1.4 to 0.1)^42^	0.31 (-0.68 to 1.29)	0.538	-
FTSST (sec)	9.9 (8.7, 12.5)	0.1 (1.0 to -0.7)^44^	0.1 (1.1 to -0.8)^44^	10.3 (9.2, 12.0)	-0.5 (-1.0 to 2.0)^45^	-0.4 (-2.0 to 2.8) [42]	0.34 (-1.26 to 1.93)	0.679	-
Nutritional status (PG-SGA SF)	1 (0, 2)	0.0 (-0.6 to 0.7)^45^	-0.6 (-1.2 to -0.05)^45^	1 (0, 3)	-0.4 (-1.1 to 0.4)^45^	-0.7 (-1.3 to -0.04)^44^	-0.4 (-1.06 to 0.27)	0.240	-
HRQOL (PDQ39 SI)	22.8 (13.5, 36.0)	-5.6 (-8.7 to -2.4)^45^	-5.7 (-8.0 to -3.4)^45^	17.8 (12.8, 27.6)	-0.2 (-2.4 to 1.99)^45^	1.3 (-0.9 to 3.6)^44^	-6.1 (-9.5 to -2.8)	< 0.001	0.93
PA frequencies	2.9 (1.8)	-	0.92 (0.3 to 1.6)^45^	3.0 (1.7)	-	-0.02 (-0.5 to 1.7)^44^	0.9 (0.2 to 1.7)	0.019	0.51

*6MWT* 6 minute walk test, measured in meters, *FTSST* Five times sit-to-stand test, measured in seconds, *PG-SGA SF* Patient-Generated Subjective Global Assessment Short Form(lower scores indicates less symptoms), *HRQOL* Health related quality of life, *PDQ39 SI* Parkinson’s Disease Questionnaire Summary index (Lower scores indicates better HRQOL) PA (Physical activity) Frequency (0-5): How often do you engage in PA? Never (0), less than once a week (1.0), 2-3 times a week (2.5) and nearly every day (5.0)

* the n varies due to missing data. Between group differences adjusted for baseline differences. Q1, Q3 = quartile 1, 3 (25th, 75th percentile). CI: confidence intervale

There was a statistically significant between-group difference in favour of the mHealth group on the PDQ-39 Summary Index. The between-group difference at 6-months, adjusted for baseline values, was mean: -6.1; 95%CI -9.5 to -2.8; *p* < 0.001; effect size = 0.93.

No other significant between-group differences were found regarding FTSST, Mini BESTest or PG-SGA SF at 6-months.

Participants in the mHealth group had significantly improved their weekly PA frequency compared to the control group from baseline to 6 months. Difference between groups mean 0.9 (0.2 to 1.7), *p* = 0.02; effect size = 0.51. Additionally, according to data from the activity tracker (Table 4), the mHealth group significantly increased their daily steps; *p* = 0.006, and weekly intensity minutes, *p* = 0.042. Since the control group did not use an activity tracker, between-group differences for this metric could not be assessed.

**Table 4:** Daily Steps and Weekly Intensity Minutes in mHealth Group

	Daily steps (N = 36) Median (Q1, Q2)	*p*-value	Weekly Intensity min (N = 36) Median (Q1, Q2)	*p*-value
1st Month	4219 (1847,6181)		2 (0,118)	
3rd Month	4426 (2318,7425)		20 (0,191)	
6th Month	5096 (2935,7887)	0.006	18 (0,518)	0.042

Measured with the Garmin Vivosmart 4. Q1, Q3 = quartile 1, 3 (25th, 75th percentile)

## Discussion

This study sought to evaluate the effect of a mHealth self-management support programme, including personalised digital self-management support on PA and nutrition, and use of an activity tracker, on physical capacity, nutritional status, HRQOL, physical function and engagement in PA in people with Parkinson’s following an inpatient interdisciplinary rehabilitation programme. The findings show that the programme significantly improved physical capacity, HRQOL and frequency of physical activity in the mHealth group compared to the control group. However, no between-group differences were found on nutritional status, balance and functional mobility after six-months.

Our positive findings on physical capacity, HRQOL and PA likely reflect the intervention’s comprehensive and individualised approach, addressing Parkinson’s specific barriers for long-term participation in health-promoting behaviours. By offering person-centred support targeting physical and behavioural factors the intervention helped people with Parkinson’s to sustain health-promoting behaviours beyond the clinical setting. These findings are consistent with recent research emphasising the limitations of single-component interventions and the importance of adopting holistic, multi-component strategies [70, 71]. A 2024 mixed methods synthesis [70] underscored the importance of broader, multi-component strategies to effectively support people with Parkinson’s. Additionally, a 2022 systematic review [71] found no consistent link between single component approaches and improvements in quality of life, well-being, or functional outcomes. Our intervention directly addressed the need for a comprehensive, person- and community-centred approach highlighted in a 2023 qualitative study [34].

Given Parkinson’s complex and fluctuating nature, a multidimensional, individualised approach is essential for supporting sustained participation. The combination of regular, personalised guidance and information, flexible communication channels, and activity tracking provided comprehensive support during the critical transition from supervised rehabilitation to independent self-management. Our results indicate that this approach may mitigate the decline in physical capacity post-rehabilitation in people with Parkinson’s. Although no significant between-group differences were detected at three months, indicating that both groups retained short-term gains from the initial inpatient rehabilitation, the significant divergence at six months, with the mHealth group improving while the control group declined in physical capacity, and only the intervention group demonstrating improved HRQOL, underscores the importance of sustained self-management support for maintaining and building on rehabilitation outcomes. While the between-group difference was smaller than estimated, the effect size was large and previous research suggest that even small improvement in walking capacity can be clinically meaningful for people with Parkinson’s [72, 73]. Notably, tailored support interventions such as our programme, offering just one hour of monthly digital support, may help reinforce rehabilitation benefits. These findings suggest a scalable and feasible supplement to more resource-intensive models, such as inpatient rehabilitation or face-to-face programmes shown to be effective in prior studies [29, 30].

A key strength in our intervention was the combined focus on PA and nutrition, a potentially synergistic approach shown to enhance outcomes in other populations [7, 74, 75], but underexplored in Parkinson’s. This integration addressed a critical gap, as inadequate energy intake can worsen fatigue, impair daily function, and limit PA benefits [76]. The lack of significant changes in nutritional status likely reflects the participants’ generally adequate baseline nutritional status. This along with the fact that only 11 participants required specialised nutritional follow-up, aligns with evidence suggesting that while issues like reduced appetite, weight loss, and malnutrition may appear early, serious reduction in nutritional status often develop more gradually and become clinically apparent in later stages [77, 78] This finding is also consistent with Ma et al. [79], who noted that nutritional interventions in Parkinson’s typically yield modest effects unless targeted at higher-risk individuals. Proactive monitoring in our study enabled early identification of the 11 at-risk participants, ensuring tailored support in accordance with existing recommendations [79]. Furthermore, our universal focus on nutrition from an early stage may help prevent or mitigate future complications, though this requires confirmation in future studies.

Sustaining guideline-based PA and nutrition is challenging for people with Parkinson’s in real-world settings, as clinical “doses” are often hard to maintain [15]. To address this, we prioritised person-centred support, helping participants to develop accessible, sustainable routines tailored to their context. This contrasts with the Park et al. study [80], whose app-based intervention, featuring exercise-diaries, smartwatch tracking and telephone counselling, found no motor symptom improvements. While they recommended the inclusion of standardised exercise programmes, our results highlight the value of individualised support with continuous adjustments. Participants receiving tailored guidance demonstrated significant increases in daily steps and weekly intensity minutes. While between-group comparisons were not possible due to lack of tracker data in the usual-care group, these within-group improvements aligned with self-reported increases in physical activity engagement, suggesting enhanced adherence to activity goals. Co-creating adaptable plans through ongoing dialog, fostered greater long-term engagement. Despite limitations in measuring PA intensity and nutritional adherence, the consistency between subjective reports and objective activity data supports our interpretation. This is further reinforced by significant between-group differences in physical capacity, underscoring the potential of personalised, flexible interventions.

Despite the observed improvements in physical activity, we found no significant between-group differences in balance or functional mobility at six-month follow-up. This may reflect the relatively high baseline scores in both groups, limiting measurable improvement, or indicate that the intervention did not sufficiently emphasize strength and balance training. Various exercise modes have shown benefits for these outcomes [81], and the flexible structure of our mHealth programme could support future iterations with greater focus on these components. Further research is needed to identify how best to integrate them into digital self-management support for people with Parkinson’s.

### Strengths and limitations

In addition to its robust RCT design, with adequate randomisation, statistical power and blinded assessors, a key strength of our study is its pragmatic, person-centred approach. By combining individualised digital consultations with wearable activity tracking, we delivered scalable and flexible support, achieving low dropout-rates (12%), high session completion (88%), and strong acceptability (82% rated support as useful or very useful).

Several limitations should be acknowledged. First, parts of the trial coincided with COVID-19 lockdowns, which may have amplified social isolation and increased the perceived value of remote support. Second, the sample’s high education level (92% tertiary level) and single-centre recruitment limit generalisability to more diverse Parkinson’s population. Third, while the chosen activity tracker provided practical, real-world insights into daily steps and intensity minutes over 6 months, it has its known limitations. Although reliable for step counting, its accuracy in measuring exercise intensity is limited, and it is not a substitute for research-grade devices [58]. Additionally, data interpretation depends on consistent wear, and many participants reported usability challenges due to its small touchscreen. Fourth, we did not collect detailed data on PA types, intensity, or nutritional adherence, nor could we compare objective activity data between groups, since the control group did not use a tracker. Finally, the absence of placebo or attention-control contacts introduces the possibility of attention bias, though the supportive interaction itself may be an integral part of the intervention efficacy.

## Conclusions

This study demonstrated that a six-month mHealth self-management support programme, providing individualised PA and nutrition guidance through monthly consultations and an activity tracker, improved physical capacity, HRQOL, and physical activity frequency in people with Parkinson’s post-rehabilitation. This pragmatic, person-centred approach enabled tailored adaptation while demonstrating transferability to diverse real-world settings, where personalised support is essential for successful implementation. The findings underscore the value of sustained, flexible self-management support. Although digital health solutions align with preferences for personalised care in Parkinson’s, limited effects on nutrition, balance and functional mobility suggest areas for further development. The findings suggest that the programme could represent a scalable and acceptable low resource mHealth model. Future strategies should expand the scope of support and focus on implementation to ensure adaptability, and equitable access.

## Supplementary Information

Supplementary Material 1.

Supplementary Material 2.

Supplementary Material 3.

Supplementary Material 4.

## Data Availability

The datasets generated and analysed during this study are all in Norwegian and not publicly available to protect participant anonymity. The data, obtained directly from participants, contain sensitive and potentially identifiable patient information. Additionally, public data sharing was not specified in the consent forms and was not part of the approved data collection terms granted by the Norwegian Agency for Shared Services in Education and Research.

## Abbreviations

mHealth: Mobile Health Technology
ICF: International Classification of Functioning
RN: Registered Nurse
H&Y: Hoehn & Yahr
PT: Physiotherapist
MoCa: Montreal Cognitive Assessment
6MWT: 6-minute Walk Test
HRQOL: Health Related Quality of Life
PDQ39: Parkinson’s Disease Questionnaire
Mini BESTest: Mini Balance Evaluation Systems Test
FTSST: Five Times Sit to Stand test
PG-SGA SF: Patient-Generated Subjective Global Assessment Short Form
PA: Physical Activity
BMI: Body Mass Index
SD: Standard deviation
ITT: Intention-to-treat
REK South/East: Regional Committee for Medical Research Ethics South/East Norway
TSD: Services for Sensitive Data
