# Supplementary material for: Post-rehabilitation self-management support on physical activity and nutrition, including mHealth, improves physical capacity, physical activity, and health related quality of life in people with Parkinson’s– results from a randomised controlled trial
Source: Int J Behav Nutr Phys Act. 2026 Feb 17;23:27. doi: 10.1186/s12966-026-01888-y (PMC13019928; doi:10.1186/s12966-026-01888-y)
Supplement: Supplementary file 2 — Supplementary Material 2. [file 12966_2026_1888_MOESM2_ESM.docx]

**Appendix1** TIDiEr CHECKLIST for mHealth intervention

| Item Nr | Item | Description | where |
| --- | --- | --- | --- |
| 1 | Name | Post-Rehabilitation Self-Management Support on Physical Activity and Nutrition, including mHealth, improves Physical Capacity, Physical Activity, and Health Related Quality of Life in People with Parkinson’s– Results from a Randomized Controlled Trial | Main article: Page 1, abstract. |
| 2 | Why | Promote and facilitate long term self-management in physical activity (PA) and nutrition for people with Parkinson’s to maintain physical function, nutritional status and quality of life. | Main article: Page 4-6, Background.  Protocol article (Alnes et al, 23), background. |
| 3 | Materials | - Phone/computer/tablet for follow-up sessions  - Smartphone (for the Garmin app)  - Garmin Vivosmart 4 for activity tracking  - Semi-structured manual (appendix 2 of protocol)  - Videoconference platforms (Zoom)  - Digital resource: Eating smart online (nutritional e. course) based on current available guidelines - [1] | Main article: Page 8-10, Intervention and Table 1. Table 1  Protocol article(Alnes et al, 23[2]), Intervention and Appendix |
| 4 | Procedures | 1. Initial face-to-face consultation: activity tracker setup, information on intervention 2. Monthly video/phone sessions (up to 60 min):  - Individualised support - Information/education - Review PA/nutritional habits - Identify barriers/motivators and ways to overcome them/ use them - Collaborative goal setting and adjustments of exercise plans  1. Text-message support between sessions 2. Nutritional specialist referrals for at-risk patients | Main article: page 8-10, Intervention and Table 1  Protocol (Alnes et al, 23[2]), Intervention and Appendix. |
| 5 | Who provided | - All follow-up sessions were delivered by a physiotherapist (PT with an MSc in Physical Therapy who contributed to developing the intervention.  - The PT delivered all sessions using a semi-structured manual  - In accordance with recommended components for self-management support, the PT used core communication skills (e.g., active listening, encouragement, empathy, and clear guidance) to support participant engagement.  - Participants identified as being at nutritional risk received follow-up from a specialist dietitian or registered nurse with a PhD in nutrition | Main article: page 10, Education of intervention deliverers |
| 6 | How | - Remote delivery: Individual Video/phone sessions  - Self-monitoring: Activity tracker  - Collaborative approach: person-centred support | Main article: page 8-10, Intervention and table 1 |
| 7 | Where | Initial setup: Rehabilitation centre  Follow-up: Participants' homes (via video/phone) | Main article: page 7, Setting and participants and Table 1 |
| 8 | When and how much | Duration: 6 months  Frequency: 6-7 sessions (1^st^ within 2 weeks post-discharged, then monthly)  Session’s length: up to 60 minutes (avg. 50 min)  Activity tracker use: everyday | Main article page: page 8-10, Intervention. Page 15, Results. |
| 9 | Tailoring | All sessions where individualised. Guidance and education based on current available guidelines, e.g. European guidelines for physiotherapy in PD[3].  - Session focus: Participant-directed priorities. Always a focus on PA and nutrition.  -PA: collaboratively develop or adjust PA plans, support and guidance adapted to individuals’ preferences, barriers and context  - Nutrition: Focus adjusted pr symptom burden  - Goals: set collaboratively based on participants interest/capabilities | Main article: page 8-10, Intervention  Protocol article: (Alnes et al, 23[2]), Intervention and Appendix |
| 10 | Modifications | No protocol modifications |  |
| 11 | How Well planned | - Protocol adherence: A third party attended 10-15% of sessions to assess protocol adherence.  - Follow-up log: number of sessions, main themes, description of how delivery was - conducted and sessions length.  - Activity tracker: log of how many used it, in which way and reasons for not using it.  Process evaluation: planned | Protocol: (Alnes et al, 23[2]), Process evaluation. |
| 12 | How well actual | Dropout: 11%  Adherence to sessions: 88% completed all monthly sessions, avg. 50 min per session.  Usefulness: 82% rated sessions useful/very useful  Activity tracker: 72%used the activity tracker for median 23 weeks (median)  Nutritional referrals: 22 % received specialist guidance | Main article: page 15, results |

1. Bye A, Kristiansen I, Støkket R, Helliesen JS. Matvett på nett Helsekompetanse.no: Norges Parkinsonforbund; Udatert [updated 2022. Available from: <https://parkinsonmatvett.vercel.app/>.

2. Alnes SR, Lærum-Onsager E, Bye A, Vistven A, Franzén E, Holst M, et al. Mobile health technology, exercise adherence and optimal nutrition post rehabilitation among people with Parkinson’s Disease (mHEXANUT) – a randomized controlled trial protocol. BMC Neurology. 2023;23(1):93.

3. Keus SHJ, Munneke M, Graziano M, Paltamaa J, Pelosin E, Domingos J, et al. European Physiotherapy Guideline for Parkinson's Disease Netherlands: KNGF/ParkinsonNet; 2014 [Available from: <https://www.parkinsonnet.com/guidelines/>.
