# Supplementary material for: Post-rehabilitation self-management support on physical activity and nutrition, including mHealth, improves physical capacity, physical activity, and health related quality of life in people with Parkinson’s– results from a randomised controlled trial
Source: Int J Behav Nutr Phys Act. 2026 Feb 17;23:27. doi: 10.1186/s12966-026-01888-y (PMC13019928; doi:10.1186/s12966-026-01888-y)
Supplement: Supplementary file 3 — Supplementary Material 3. [file 12966_2026_1888_MOESM3_ESM.docx]

**Appendix 2** Intervention alignments with self-management frameworks

| **Lorig & Holman Self-Management skills[1]** | **Dineen-Griffin et al [2] Key Self-Management Support Components** | **Intervention integration** |
| --- | --- | --- |
| Problem solving | Structured patient-provider exchange: One-on-one consultation, ongoing follow-up, self-help materials. | Monthly individualised consultation (video/phone) provided a structured platform for participants to identify and discuss personal, social and environmental barriers to adherence. The follow-up PT supported participants in generating and evaluating solutions to these problems. |
| Decision making | Tailored interventions: strategies to improve disease/treatment knowledge | Guidance was aligned with current clinical guidelines. Participants received information and support to make informed decisions regarding exercise intensity, managing symptoms affecting activity, and nutritional choices. |
| Resource utilisation | Structured patient-provider exchange: provision of self-help materials. | The intervention included recommended referrals to other healthcare personnel (e-g- nutritional specialist) when necessary. Participants were encouraged to utilise the activity tracker as a self-monitoring tool, providing real-time feedback. Digital resources like 'Eating smart online'[3] were also suggested. |
| Patient-provider partnership | Collaborative partnership: patient-centeredness enhancing responsibility. | The intervention was explicitly designed around a person-centred, collaborative approach. The follow-up PT fostered a partnership by encouraging reflections, collaboratively setting goals, adapting plans based on individual progress, and allowing participants significant autonomy in directing conversations. |
| Action planning | Independent monitoring and personalised action plan: encouraging self-treatment through a personalised plan. | Participants collaboratively set realistic and motivation goals and developed action plans based on their interests and capabilities. The activity tracker facilitated self-monitoring of daily activity and exercise intensity, supporting the implications of these plans. |
| Medical management | Independent monitoring of symptoms: encouraging self-treatment, enhancing responsibility in medication adherence and lifestyle choices. | The intervention addressed exercise and nutrition, which are key components of medical management for PD. Discussions included managing symptoms affecting intake (e.g. constipation, swallowing difficulties) and planning meals to avoid food-medication interaction. The PT also encouraged talking to a neurologist if they had questions on medication adjustments, or symptom changes and fluctuations that may warrant a reassessment of medication. |
| Role management | Psychological coping and stress management | Discussions around managing daily energy balance and addressing barriers related to sleep, pain, or medication management implicitly supported participant in maintain life roles. |
| Emotional management | Psychological coping and stress management | The intervention aimed to build self-efficacy and positive outcome expectations, which can support emotional well-being. Discussions around pain and sleep disturbances, and having someone to talk to about general disease-related concerns is also touch upon the emotional aspects of living with PD. |

1. Lorig KR, Holman HR. Self-management education: history, definition, outcomes, and mechanisms. Annals of behavioral medicine. 2003;26(1):1-7.

2. Dineen-Griffin S, Garcia-Cardenas V, Williams K, Benrimoj SI. Helping patients help themselves: A systematic review of self-management support strategies in primary health care practice. PloS one. 2019;14(8):e0220116.

3. Bye A, Kristiansen I, Støkket R, Helliesen JS. Matvett på nett Helsekompetanse.no: Norges Parkinsonforbund; Udatert [updated 2022. Available from: <https://parkinsonmatvett.vercel.app/>.
